# A mouse-tracking classification task to measure the unhealthy = tasty intuition

**DOI:** 10.3758/s13428-025-02927-5

**Published:** 2026-01-16

**Authors:** Jonathan D’hondt, Barbara Briers

**Affiliations:** 1 Department of Marketing, Vrije Universiteit Amsterdam, De Boelelaan 1105, 1081 HV Amsterdam, Netherlands; 2 Department of Work, Organisation and Society, Ghent University, Henri Dunantlaan 2, 9000 Ghent, Belgium; 3 Vlerick Business School, Area Marketing, Reep 1, 9000 Ghent, Belgium; 4 Department of Marketing, University of Antwerp, Prinsstraat 13, B2000 Antwerp, Belgium

**Keywords:** Mouse tracking, Measurement, Food lay beliefs, Unhealthy = tasty intuition

## Abstract

Understanding food preferences plays a crucial role in addressing both health concerns, such as obesity, and environmental concerns, such as climate change. Recognizing the impact of lay beliefs on food preferences is essential in addressing these challenges. One prevalent belief is the “unhealthy = tasty intuition” (UTI), the belief that taste and health in food do not go together. While self-report scales and behavioral tasks are commonly used to measure such beliefs, they have distinct methodological purposes: scales are better suited for assessing stable, trait-like constructs, whereas tasks capture more dynamic processes and are well suited for experimental manipulation. This paper introduces a mouse-tracking classification task that provides a process-based behavioral index of UTI, providing a novel approach for assessing implicit beliefs about the relationship between taste and health in food. Three studies validate the task, demonstrating correlations between explicit UTI scores and task performance. Additionally, the task predicts actual food consumption and, importantly, exhibits sensitivity to contextual manipulations. Because this task can be adapted to measure other beliefs, it is a valuable tool for researchers working on individual lay beliefs and decision-making processes. To that end, a template of the task is provided to help other researchers build on this work.

## Introduction

Food takes center stage in two of our society’s biggest challenges: the way we eat contributes to health concerns, such as the rising obesity epidemic (e.g., Jebb, 2004), and is a significant factor in environmental problems, such as climate change (e.g., Kroyer, 1995). Researchers have long been interested in the complexities of food choices, driven by a commitment to understanding and addressing these critical issues that impact people worldwide.

A particular area of interest is how food-related lay beliefs impact our eating behaviors. Lay beliefs can significantly impact eating behaviors by shaping individuals' perceptions, attitudes, and food choices. For example, people who believe obesity is caused by a lack of exercise tend to eat and weigh more than people who believe obesity is caused by diet (McFerran & Mukhopadhyay, 2013). Another important belief is the “unhealthy = tasty intuition” (UTI; Raghunathan et al., 2006). People who strongly adhere to this belief experience a conflict between taste and health in food, resulting in an unhealthier diet and an increased risk for being overweight (Briers et al., 2020; Cooremans et al., 2017).

The measurement of psychological constructs such as lay beliefs is not straightforward. In psychology, self-report scales and behavioral tasks are the most popular measurement methods. Self-report measures, often called “explicit,” are useful when individuals have a clear understanding of the construct being studied and can assess their own feelings and thoughts (Paulhus & Vazire, 2007). They are efficient for screening large groups and are suitable for understanding someone's current state or general trends over time. A common critique of self-report scales, however, is their susceptibility to response biases, including social desirability bias. Participants may provide answers that they believe are socially acceptable or that align with societal norms rather than their true thoughts or behaviors (e.g., Paulhus, 1991). To counter these potential shortcomings, researchers often employ behavioral tasks that measure the target construct indirectly or under conditions of automaticity (e.g., with strict time constraints), often called “implicit.” Behavioral tasks usually require the participants to make a series of decisions in a repeated-measures design. A well-known example is the implicit association task (IAT; Greenwald et al., 1998), in which participants are asked to rapidly classify stimuli into categories. The test is thought to measure the strength of automatic associations between concepts.

Another way to categorize measurement methods is by their research objectives, as outlined by Deltomme (2023). These objectives include (1) exploring individual differences and (2) examining situational influences. When assessing individual differences, researchers seek stability unaffected by situational influences, making self-report scales the optimal choice. In contrast, for examining the impact of situational influences in experiments, researchers prefer measures that are less stable, best resorting to behavioral tasks. Self-report scales have higher temporal stability and thus are less sensitive to (experimentally) induced situation changes, making them less ideal for experimental research (e.g., Gawronski et al., 2017). Conversely, behavioral tasks such as the IAT are often reported to have low reliability (e.g., Aberson & Beeney, 2007; Rae & Olson, 2018; Schmukle & Egloff, 2005), which renders them less suitable for measuring individual differences. Despite their low reliability, or rather because of it, these types of measures have a different utility. Their sensitivity to within-person experimental changes makes them valuable for investigating underlying processes and the contextual factors influencing the construct of interest (Brunel et al., 2004). However, researchers tend to use these methods interchangeably for the same construct, disregarding the distinct purposes of self-report and behavioral tasks (Dang et al., 2020), which has, to some extent, put the IAT in a bad light.

Overall, the IAT has been criticized with regard to its validity and the interpretation of its results (e.g., De Houwer et al., 2009). First, the IAT is a rather complex and cognitively demanding task (Brunel et al., 2004). Second, the index generated by the IAT represents the outcome of a cognitive process but provides no information about its dynamic properties. While reaction times do carry information about cognitive conflict, they are influenced by multiple overlapping factors, such as perceptual delays and accuracy goals, which can obscure the specific contribution of conflict (Gawronski et al., 2007; Stillman et al., 2018). Instead, we propose an alternative behavioral task based on mouse-tracking, an increasingly popular method in the behavioral sciences. Mouse-tracking is a technique that tracks the computer cursor at high temporal resolution as participants select an alternative on the screen (Freeman, 2018) and has the benefit of allowing the unfolding of the response to be tracked during the trial. This results in richer information about *how* the decision was made, for example, indicating to what extent there was a conflict between possible responses and how that conflict was resolved in real time (Stillman et al., 2018). In addition, mouse-tracking hardware is inexpensive, software is freely accessible, and most people are familiar with using a mouse, making them less likely to be aware that their movements are being tracked. Mouse-tracking has been used, for example, in studies assessing how branding influences consumers’ choices (Fisher & Woolley, 2024) and in identifying racial biases (Melnikoff et al., 2021). These studies are based on theoretical frameworks that emphasize continuous, dynamic, real-time thinking (e.g., Dotan et al., 2019; Freeman & Ambady, 2010).

Unlike traditional views in cognitive psychology, which see human thinking and behavior as occurring in distinct stages, dynamic cognitive frameworks suggest that thinking and movement unfold as an ongoing, changing series of brain activities. Consequently, motor movements are not launched once cognitive processing is finished but are continuously adjusted to mirror ongoing cognitive processing dynamics (see Freeman, 2018, for a review). Some researchers have used this idea in previous studies to examine the cognitive processing of food and food choices. For example, Sullivan et al. (2015) found that taste, as a basic attribute, is generally processed faster than healthiness, a more abstract attribute. Similarly, Ha et al. (2019) used mouse-tracking to show that rejecting unhealthy foods requires more cognitive effort than accepting them.

### Mouse-tracking to measure UTI

Individuals who strongly adhere to the UTI demonstrate a strong association between the taste and health attributes of food. For these individuals, the perceived taste of food is linked to its perceived healthiness. On the contrary, individuals who do not adhere to the UTI do not exhibit such an association. For them, taste perception remains independent of the health classification of food. The UTI is mainly measured with a self-report scale or the IAT (e.g., Raghunathan et al., 2006). We add to the literature by introducing a mouse-tracking classification task that provides a process-based behavioral index of UTI. In this task, participants view images of typically (un)healthy food and are prompted to classify them as either “tasty” or “not tasty.” We will compare the difference in classification speed and trajectory between unhealthy and healthy food items among individuals with higher versus lower UTI beliefs. Individuals with a higher UTI, who tend to see a direct correlation between tastiness and healthiness of food, should show a systematic difference in the classification of response patterns for healthy and unhealthy food items. Conversely, individuals with a lower UTI, who do not see a direct correlation between food tastiness and its healthiness, should show no difference in their responses to healthy and unhealthy food items. Specifically, we predict that the difference in classification speed between unhealthy and healthy food will be larger for individuals with stronger versus weaker UTI beliefs. We interpret these differences as reflecting the extent to which the UTI is engaged during evaluation. Furthermore, we will explore whether the relationship between UTI and motor behavior during the classification task is choice-contingent. Specifically, individuals with stronger UTI beliefs could show greater conflict (i.e., more complex cursor trajectories) when their response runs counter to their intuitive association—such as when they classify a healthy food as “tasty.” In contrast, for these same individuals, categorizing healthy food as “not tasty” or unhealthy food as “tasty” should involve less conflict, possibly resulting in more direct, fluent trajectories.

We test this in three studies. Studies 1 and 2 compare self-reported UTI and UTI expressed in the classification task under stable conditions. In Study 3, we manipulate goal orientation to test whether the task is sensitive to contextual factors. Materials and preregistrations for all studies are available on https://osf.io/qej3k/overview.

## Study 1

In this study, we examined the correlation between the self-report scale and mouse-tracking outcomes. Although self-report measures and behavioral tasks are typically suited for different research objectives—specifically, measuring stable individual differences and effects of situational influences, respectively (e.g., Deltomme, 2023)—they are not entirely independent and should show some overlap. In their meta-analysis, Hofmann and colleagues (2005) found an average correlation of .24 between explicit and implicit measures in general. Study 1, therefore, assessed whether individuals’ explicit endorsement of the UTI corresponds with their behavioral processing costs when classifying healthy versus unhealthy foods as tasty or not tasty.

### Participants

We recruited 200 UK participants on Prolific who were paid £1 for a task estimated to take 5 min. Participants were on average 41.06 years old (*SD*_Age_ = 13.70); 60% of the participants were female. Only participants who used a desktop computer with an external mouse and who were right-handed were allowed to participate. All participants provided informed consent prior to participation. We conducted a power analysis using G*Power (Faul et al., 2007) to determine the minimum sample size required for the correlation analysis. Based on an expected correlation of *r* = .24, as reported in previous literature (Hofmann et al., 2005), with a desired power level of 90% and an alpha level of .05, the analysis indicated that we would need a minimum of 142 participants. Therefore, the recruited sample size of 200 participants ensures adequate statistical power to detect the hypothesized effect.

### Method

The study was coded in lab.js (Henninger et al., 2022). Participants were provided with the explicit UTI scale and the UTI classification task. We counterbalanced the order of the tasks.

The self-report UTI scale was a three-item, seven-point Likert scale (Raghunathan et al., 2006; α = .94; 1 = Strongly disagree, 7 = Strongly agree). The items were “Healthy food is usually less tasty,” “Eating healthy means sacrificing taste,” and “There is usually a trade-off between healthiness and tastiness of food,” and item scores were averaged.

The behavioral classification task is a computer task in which participants classified food items as “tasty” or “not tasty” by moving the mouse and clicking the response boxes in the upper-left and upper-right corners, respectively. Trials started by moving the cursor to a button at the bottom center of the screen. We employed a dynamic starting procedure, meaning that participants had to initiate their response movement to trigger the food stimulus presentation.^1^ Stimuli comprised 10 healthy and 10 unhealthy food items, selected from the publicly available *food-pics_extended stimulus* database (Blechert et al., 2019). All images were rated at least 80 out of 100 on average on familiarity in the original database and were standardized in size. All images were shown twice for a total of 40 trials. In a post-test, we confirmed that our selected stimuli were representative of their respective category, i.e., (un)healthy food items were perceived as (un)healthy (see Appendix B on https://osf.io/qej3k/overview). Before the task, participants received instructions and a short practice block of six trials to familiarize themselves with the procedure.

Cursor tracking was conducted using the Mousetrap plugin, which offers a tracking resolution of 100 Hz, meaning the cursor position was recorded every 10 ms. Tracking started when participants removed the cursor from the starting button and ended upon clicking one of the response boxes. Following established guidelines from prior research (Dotan et al., 2019), we applied a transformation to shift and normalize coordinates. Specifically, the point where the cursor exited the start box was designated as (*x*, *y*) = (0, 0), the pixel clicked to select the left option was mapped to (−1, 1), and the pixel clicked to choose the right option was mapped to (1, 1). Participants were instructed to (1) respond naturally by moving the cursor continuously from the start button toward the top side of the screen of the desired choice option and (2) respond as quickly as possible.

### Dependent variables

#### Reaction times

The time from the start of cursor movement to the time participants clicked the response box was measured in milliseconds (ms). To test our hypothesis, we calculated reaction times for unhealthy and healthy food trials separately and then computed a difference score, with higher values indicating slower reaction times to healthy foods. Reaction times slower and faster than three standard deviations from the mean were excluded from the analysis (1.4% of trials), as preregistered (e.g., Berger & Kiefer, 2021). In addition to the preregistered participant-level difference-score approach, we also fitted a trial-level linear mixed-effects model to confirm that the effect held when every trial and its covariates were included.

#### Maximum absolute deviation

**Fig. 1:**
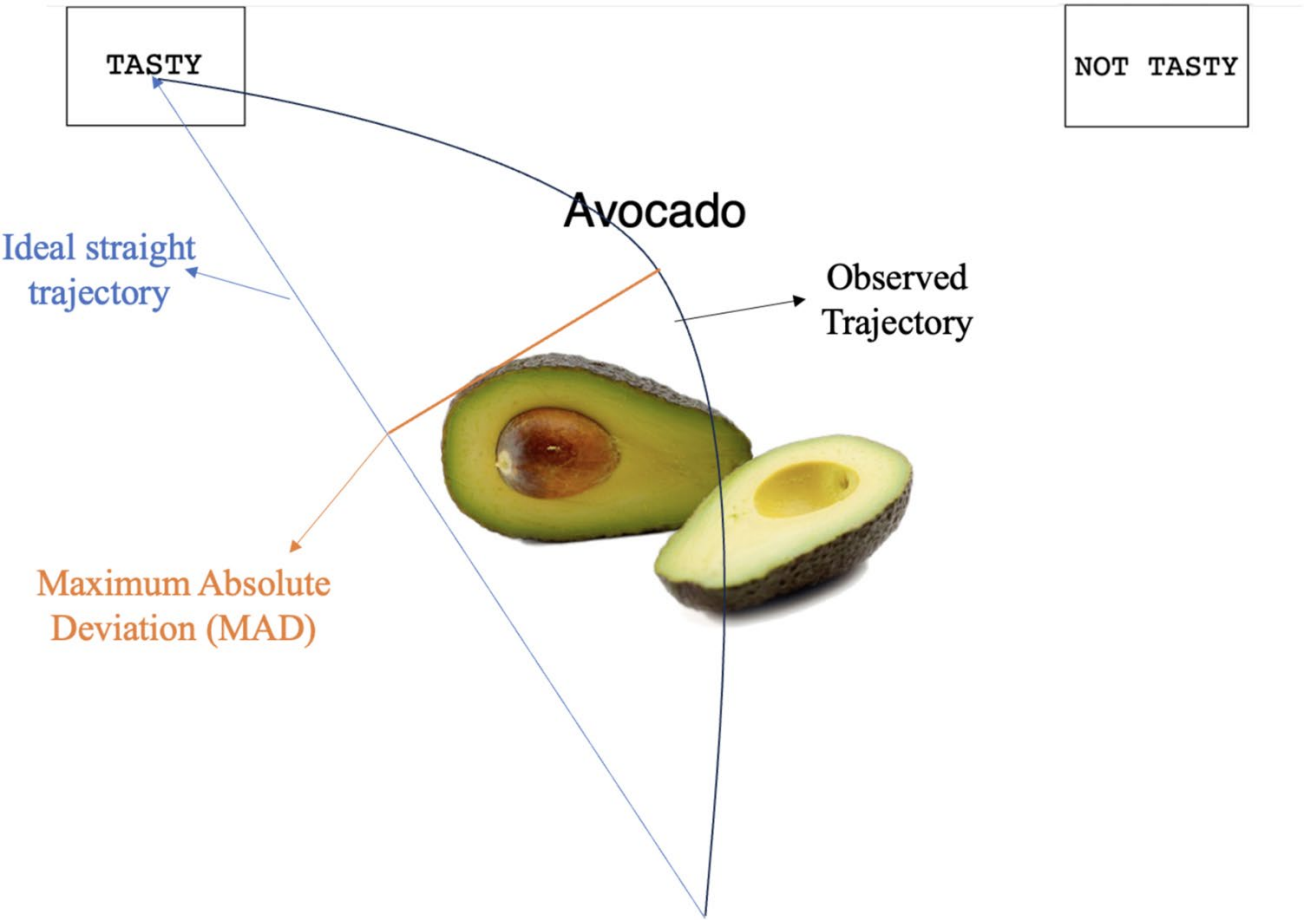
An example of a trial in the classification task and a representation of the MAD. The blue line represents the most efficient response path from start to finish. The black line shows an observed response path. The orange line illustrates the MAD, the largest perpendicular distance between those paths

Cursor trajectories were spatially normalized using 101 equidistant points (i.e., 0–100% of the movement distance) along the original cursor trajectory. The signed maximum absolute deviation (MAD; see Fig. 1) represents the highest perpendicular deviation (px) from the straight trajectory connecting the initial and final points of a trajectory (Freeman & Ambady, 2010). Trajectories with higher MAD are typically interpreted as reflecting greater competition between response alternatives and, by extension, higher decisional conflict (Freeman, 2018; Stillman et al., 2018). Again, we calculated the MAD for the unhealthy and healthy food trials separately and then took the difference score, so that higher positive values indicate less efficient reactions to healthy foods. As with the reaction time variable, we preregistered to exclude trials in which the MAD exceeded three standard deviations from the mean (1.2% of trials).

### Results

#### Classification distribution

Participants classified the large majority of foods as tasty, regardless of whether the item was healthy or unhealthy. Across the full sample, 75% of healthy items and 83% of unhealthy items were classified as “tasty,” leaving 25% and 17% as “not tasty,” respectively. For descriptive purposes, we examined this distribution after a median split on explicit UTI. In the low-UTI group, approximately 80% of both healthy and unhealthy items were classified as “tasty,” leaving ~20% “not tasty” classifications for each food type. In the high-UTI group, ~65% of healthy foods were classified as “tasty” (35% “not tasty”), whereas ~85% of unhealthy foods were classified as “tasty” (15% “not tasty”).

This asymmetry is important for interpreting the preregistered difference-score dependent variables (healthy–unhealthy). Because the majority of trials were classified as tasty, the difference scores are dominated by responses in which participants endorsed foods as tasty. As such, they primarily capture the relative processing cost of classifying healthy versus unhealthy items as tasty. As a result, the preregistered healthy–unhealthy difference score index captures the most diagnostic cases: how much harder (or easier) it is for individuals to classify healthy foods as tasty compared to unhealthy foods, which we will rely on to show that people with a stronger UTI have a harder time liking healthy foods. At the same time, collapsing across classifications risks obscuring conflict effects on the less frequent “not tasty” trials. To address this, we implemented classification-integrated analyses at the trial level that account for both food type and overt classification, providing a more complete characterization of how UTI relates to processing costs.

For descriptive purposes, trajectories and statistics broken down by food type, classification, and UTI group are provided in Appendix C, along with additional exploratory analyses.

#### Preregistered difference scores

**Reaction times.** We found a significant positive correlation between the explicit UTI scale measure and the reaction time difference score, *r* = .29, *p* < .001. This indicates that participants with higher explicit UTI scores were slower to categorize healthy food items than unhealthy ones. We did not find an effect of task order on reaction time difference, *t*(195.17) = −.61, *p* = .540.

**MAD.** We found a significant positive correlation between the explicit UTI scale measure and the MAD difference score, *r* = .32, *p* < .001. This indicates that participants with a higher explicit UTI were less efficient at categorizing healthy food items than unhealthy food items. Here again, we did not find an effect of task order, *t*(185.1) = 1.34, *p* = .183.

**Fig. 2:**
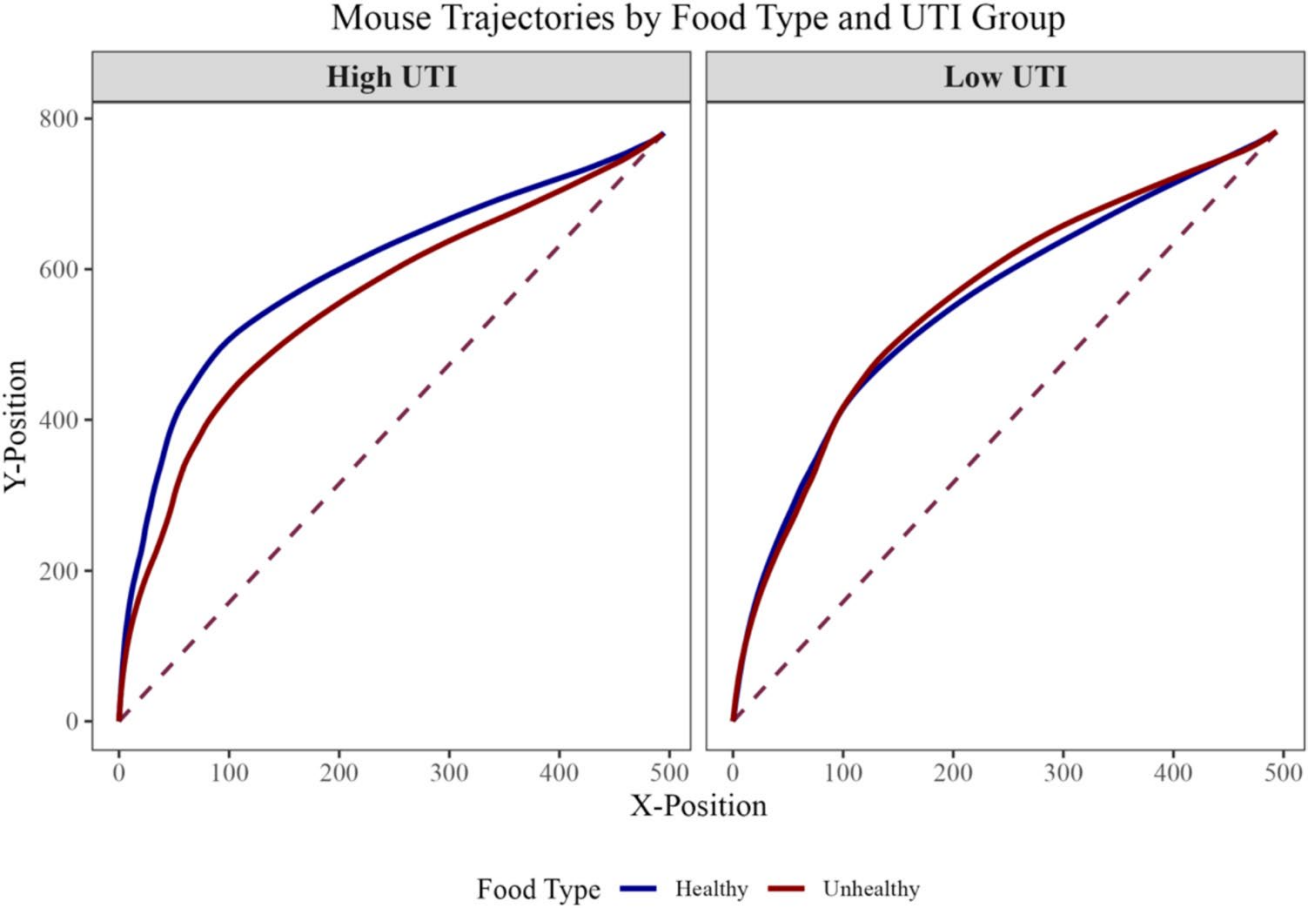
Visual representation of response trajectories of healthy and unhealthy food items for participants low and high on the explicit UTI scale after a median split

To visualize these differences in motor dynamics, we plotted the average cursor trajectories for healthy and unhealthy food items, split by high versus low UTI groups after a median split (Fig. 2) for descriptive purposes. In the high UTI group, the trajectories for healthy versus unhealthy items diverged early in the movement, indicating a less efficient trajectory for healthy food classification. Conversely, the low UTI group showed largely overlapping trajectories for healthy and unhealthy items, with minimal differentiation in cursor movements.

#### Exploratory analyses

**Reaction times.** To test whether these correlations held at the trial level and when accounting for covariates, we fit a linear mixed-effects model predicting RT from food type (healthy vs. unhealthy), classification (“tasty” vs. “not tasty”), explicit UTI, and their interactions, controlling for age, gender, and education, with random intercepts for participant and food item. The model revealed main effects of classification (slower responses for “not tasty” trials, β = 149.149, *SE* = 27.49, *p* < .001), UTI (higher-UTI participants were slower overall, β = 32.05, *SE* = 8.97, *p* < .001), and age (β = 9.10, *SE* = 1.05, *p* < .001). Crucially, the UTI × Food Type interaction was significant (β = −14.40, *SE* = 4.46, *p* = .001), indicating that the positive association between UTI and response latency was strongest for healthy trials. A UTI × Classification effect (β = −14.29, *SE* = 7.08, *p* = .040) suggests that the positive relationship between UTI and RT is weaker for “not tasty” trials, or in other words, that high-UTI participants’ slowing is primarily evident on “tasty” trials. The three-way interaction did not reach significance (β = 19.05, *SE* = 10.12, *p* = .060). Together, these results indicate that explicit UTI robustly predicts slower responses to healthy foods. The trend toward a three-way interaction raises the possibility that this effect may be especially pronounced when healthy items are judged as tasty. Still, the present sample, with relatively few “not tasty” responses, does not conclusively demonstrate this.

**MAD.** We also fit a linear mixed-effects model predicting MAD from food type (healthy vs. unhealthy), classification (“tasty” vs. “not tasty”), explicit UTI, and their interactions, controlling for age, gender, and education, with random intercepts for participant and food item. Results revealed main effects of classification (greater curvature for “not tasty” responses, β = 189.33, *SE* = 19.39, *p* < .001) and UTI (higher-UTI participants responded with more curved trajectories overall, β = 12.23, *SE* = 4.63, *p* = .009). Crucially, the UTI × Food Type interaction was significant (β = −7.45, *SE* = 3.07, *p* = .015), indicating that the positive association between UTI and curvature was strongest for healthy trials. Neither the UTI × classification interaction nor the three-way interaction reached significance, suggesting that the UTI effect did not depend on the specific classification made. In other words, individuals who more strongly endorsed the unhealthy = tasty intuition consistently showed greater motor inefficiency when evaluating healthy foods, regardless of whether they ultimately classified them as tasty or not tasty.

#### Choice artifact

One concern is that UTI-related costs on healthy items might simply reflect a greater prevalence of “not tasty” responses among high-UTI participants. Because “not tasty” responses are generally slower and more curved for all participants (consistent with previous literature such as Ha et al., 2019), an overrepresentation of these trials could artificially produce the appearance of a UTI × Food Type effect. To test this possibility, we estimated simple slopes of UTI within each Food Type × Classification cell from the trial-level mixed models.

Results showed that within “tasty” responses, the UTI slope was reliably steeper for healthy than unhealthy items for RT (Δ = 24.06 ms per 1 *SD* UTI, *SE* = 7.46, *z* = 3.23, *p* = .001) and MAD (Δ = 12.50 px, *SE* = 5.12, *z* = 2.43, *p* = .015). In contrast, within “not tasty” responses, the healthy–unhealthy difference in the UTI slope was not significant for any metric (all *p*s ≥ .30). Thus, explicit UTI predicts disproportionate increases in processing cost on healthy items even when the response is “tasty,” indicating that the effect is not reducible to a greater frequency of “not tasty” responses among high-UTI participants.

### Discussion

In our first study, we found initial evidence that the behavioral task indices correlate with the explicit UTI measure. These correlations are not large but are in line with the literature (Hofmann et al., 2005). Furthermore, we found comparable findings with a different set of food images and a static starting procedure in a replication study available on https://osf.io/qej3k/overview.

Importantly, the trial-level mixed-effects models provided a clearer picture of how explicit UTI relates to food classifications and motor dynamics. As expected, participants with higher UTI scores were less likely to classify healthy foods as tasty, confirming that the scale reflects a negative bias against linking healthiness with tastiness. Beyond this choice shift, however, higher UTI also predicted disproportionate processing costs on healthy items: slower reaction times and greater trajectory curvature (MAD). Simple slope analyses showed that these healthy-specific UTI effects were evident within tasty trials, indicating that they cannot be explained solely by high-UTI individuals producing more “not tasty” responses, which were generally harder for everyone. In contrast, UTI effects were weaker and less reliable in “not tasty” trials, likely because of their lower frequency. Together, these findings suggest that explicit UTI is reflected not only in what people choose, but also in how they enact those choices. Thus, Study 1 provides converging evidence that individuals who endorse the unhealthy = tasty intuition both (a) show a behavioral tendency to reject healthy foods as tasty and (b) display greater cognitive–motor conflict when evaluating healthy items, with the pattern being directionally strongest when participants affirmed those items as tasty. These results strengthen the construct validity of the UTI classification task by demonstrating that explicit beliefs are mirrored in continuous motor measures of conflict during decision-making.

In the second study, our goals were to (1) replicate the findings of the first study and (2) use the task to predict a downstream consequence of UTI: food consumption. People who adhere strongly to the UTI consume fewer vegetables on average (Briers et al., 2020). While the predictive validity of behavioral tasks is often the topic of debate (e.g., Brownstein et al., 2019) and their incremental validity over explicit measures is found negligible (Meissner et al., 2019), the implicit measure should at least point in the same direction as the explicit measure to demonstrate content validity.

## Study 2

### Participants

The planned sample size was determined a priori based on the smallest effect observed in a pilot study (correlation between UTI and RT difference, *r* = .21). A Fisher *z* power analysis indicated that *N* = 235 analyzable participants would provide 90% power to detect this effect at α = .05 (two-tailed). To allow for potential exclusions due to device noncompliance or incomplete questionnaires, we recruited 250 Prolific participants. If the number of usable cases had fallen below the preregistered threshold of 235, we planned to collect additional data to reach that target.

Three participants were excluded for incomplete data, leaving a final sample of *N* = 247 (*M*_age_ = 48.0, *SD*_age_ = 15.1; 47% female). Eligibility criteria matched Study 1: right-handed, fluent English speakers using a desktop computer with an external mouse. All participants provided informed consent before participation.

### Method

The study followed the procedure of Study 1, adding a food frequency questionnaire (FFQ) at the end of the procedure. Participants were asked about the consumption frequency of 20 food categories (e.g., vegetables, processed meats; 1 = “Rarely or never,” 8 = “5+ times a day”). The scores for all food items were combined into a single diet quality score (DQS) according to the original authors’ guidelines (Cleghorn et al., 2016). Scores could range from 5 to 15, with a higher score representing a healthier diet. Previous research has demonstrated that poorer diet quality is a downstream consequence of the UTI, linking stronger UTI beliefs to, on average, less healthy eating behaviors, especially lower vegetable intake (e.g., Briers et al., 2020; Cooremans et al., 2017).

### Preregistration and analytic plan

All hypotheses, variables, and exclusion criteria were preregistered before data collection (see https://osf.io/qej3k/overview). The preregistered analyses closely followed those of Study 1. Specifically, we predicted that higher explicit UTI scores would be associated with slower responses and greater curvature of the mouse trajectory for healthy relative to unhealthy foods (ΔRT and ΔMAD). As in Study 1, we fit linear mixed-effects models for both RT and MAD at the trial level, this time preregistered. Each model included the fixed factors explicit UTI, food type (healthy vs. unhealthy), and taste classification (“tasty” vs. “not tasty”), as well as all lower-order and three-way interactions among these factors. Participant age, gender, and education were included as covariates. Random intercepts for participant and food item were specified to account for repeated observations within subjects and stimuli. Based on the results of Study 1, the key preregistered prediction was a significant UTI × Food Type interaction, indicating that higher UTI would predict slower and more curved responses for healthy relative to unhealthy foods. As preregistered, trials with reaction times or MAD larger than 3 SDs (1.4% and 1.7%, respectively) were removed from the analyses.

Finally, we predicted that stronger UTI beliefs (both the explicit score and classification task indices) would correspond to poorer diet quality, as measured by the diet quality score (DQS). In exploratory analyses, we examined whether the mouse-tracking indices (ΔRT, ΔMAD) explained unique variance in DQS beyond explicit UTI scores.

As in Study 1, participants classified the large majority of foods as tasty, with only modest differences between healthy and unhealthy items. Across the full sample, 71% of healthy items and 82% of unhealthy items were judged as tasty. For descriptive purposes, we again examined this distribution after a median split on explicit UTI. In the low-UTI group, 76% of healthy items and 77% of unhealthy items were judged as tasty, whereas in the high-UTI group, 67% of healthy items and 87% of unhealthy items were judged as tasty. Thus, as in Study 1, participants’ choices were dominated by “tasty” responses, supporting the use of difference scores (healthy–unhealthy) as dependent variables in subsequent analyses.

### Results

#### Replication of Study 1 effects

As predicted, explicit UTI scores correlated positively with both RT and MAD difference scores (*r*s = .33 and .30, respectively; both *p* < .001). Participants who more strongly endorsed the belief that unhealthy foods taste better were slower and exhibited greater trajectory curvature when categorizing healthy foods, consistent with increased decisional conflict.

Trial-level mixed-effects models confirmed the preregistered UTI × Food Type interaction for both outcome measures.

For reaction times, β = −19.17, *SE* = 3.75, *t*(9371) = −5.12, *p* < .001, indicating that higher UTI scores predicted slower responses for healthy than for unhealthy foods. In contrast to Study 1, the three-way UTI × Food Type × Classification interaction reached significance, β = 33.30, *SE* = 8.70, *t*(9453) = 3.91, *p* < .001, showing that the health-specific slower responses were especially pronounced when participants judged foods as tasty.

The MAD model yielded the same pattern: a significant UTI × Food Type interaction, β = −7.76, *SE* = 2.51, *t*(9428) = −3.09, *p* = .002, and a three-way interaction with classification, β = 15.06, *SE* = 5.80, *t*(9548) = 2.60, *p* = .009. Thus, individuals higher in UTI displayed greater trajectory curvature for healthy foods, particularly when those foods were labeled tasty. These results replicate the core behavioral effects from Study 1 and confirm the preregistered hypotheses. Full results for both models are available in Appendix C.

To assess whether UTI-related costs on healthy items could be attributed to a greater prevalence of “not tasty” responses among high-UTI participants, we again estimated the simple slopes of UTI within each Food Type × Classification cell.

The results replicated the pattern observed in Study 1. Within “tasty” responses, the UTI slope was significantly steeper for healthy than unhealthy items for both RT (Δ = 19.17 ms per 1 *SD* UTI, *SE* = 3.75, *z* = 5.12, *p* < .001) and MAD (Δ = 7.76 px, *SE* = 2.51, *z* = 3.09, *p* = .011). In contrast, within “not tasty” responses, the corresponding healthy−unhealthy differences were nonsignificant (all *p*s > .20).

Thus, explicit UTI again predicted disproportionate increases in processing cost on healthy–tasty items, indicating that the effect is not reducible to a greater frequency of “not tasty” responses among high-UTI participants.

### Associations with diet quality (DQS)

Explicit UTI scores correlated negatively with DQS (*r* = −.33, *p* < .001), indicating that participants who more strongly endorsed the UTI reported less healthy diets. Both behavioral indices also correlated negatively with DQS (ΔRT: *r* = −.17, *p* = .009; ΔMAD: *r* = −.25, *p* < .001), such that slower RT or curvature for healthy foods correlated with poorer diet quality.

To examine incremental predictive validity, we regressed DQS on explicit UTI and either ΔRT or ΔMAD while controlling for age, gender, and education. Both predictors were significant (β_UTI_ = −.27, *t*(238) = −4.53, *p* < .001; β_ΔMAD_ = −.003, *t*(238) = −2.38, *p* = .005). A nested model comparison confirmed that adding ΔMAD significantly improved prediction beyond UTI alone, Δ*F*(1, 240) = 7.92, *p* = .005. In contrast, ΔRT was not a significant predictor (β_ΔRT_ = −.001, *t*(238) = −1.26, *p* = .208). Thus, the mouse-tracking curvature index, but not response time, captured unique variance in diet quality beyond explicit beliefs.

### Discussion

Study 2 replicated and extended the findings of Study 1 within a preregistered framework. Consistent with our first goal, the mouse-tracking classification task again revealed robust UTI effects: participants who more strongly endorsed the UTI belief were slower and exhibited more conflicted cursor movements when evaluating healthy foods. These effects were selective to healthy–tasty judgments, matching the pattern observed in Study 1, and were obtained using preregistered mixed-effects models that controlled for demographic covariates.

Consistent with our second goal, the implicit indices also predicted a meaningful downstream outcome: self-reported diet quality. Both ΔRT and ΔMAD correlated negatively with the DQS, indicating that participants who showed greater processing conflict for healthy foods tended to report less healthy eating habits. Importantly, the curvature-based index (ΔMAD) accounted for unique variance in diet quality beyond explicit beliefs, providing evidence for incremental predictive validity. This suggests that dynamic motor signatures of conflict can capture aspects of the UTI that are not fully accessible to the self-report questionnaire.

Taken together, the results demonstrate that the mouse-tracking classification task provides a robust and sensitive measure of implicit unhealthy = tasty associations. The task replicated the characteristic pattern of greater decisional conflict for healthy foods and, critically, these behavioral indices were linked to self-reported diet quality. This predictive association supports the task’s validity as a process-based behavioral measure that complements self-report, showing its potential for studying implicit evaluative biases that underlie everyday food judgments.

Together, the first two studies established the reliability and predictive validity of the mouse-tracking classification task as a measure of implicit UTI. The next step was to examine its experimental sensitivity: whether the task can detect momentary shifts in these associations when situational goals change.

## Study 3

In this study, we wanted to test the suitability of the mouse-tracking classification task for experimental studies. Behavioral tasks are sensitive to short-term changes in how associations are activated in memory (Gawronski & Bodenhausen, 2006, 2011). This sensitivity could be due to contextual cues and situational factors shaping associations differently each time we measure them. When it comes to food choices, contextual factors shape which food attributes weigh more heavily in the decision-making process. For instance, directing attention toward the healthiness of food influences cognitive processing and encourages healthier food choices (Hare et al., 2011).

In contrast, explicit self-report measures capture what a person believes to be true or false, reflecting more stable outcomes from reasoning processes. This distinction makes behavioral tasks more suitable for studies using contextual manipulations. In this study, we therefore manipulated a hedonic versus utilitarian goal orientation using the same procedure as the original UTI paper (Raghunathan et al., 2006). We know from previous research that when consumers face the task of choosing among food products, options perceived as better-tasting are more likely to be chosen when a hedonic goal is more salient (e.g., Dhar & Simonson, 1999; Shiv & Fedorikhin, 1999). Extrapolating from these findings, Raghunathan et al. (2006) proposed and demonstrated that participants will prefer options portrayed as more unhealthy under a salient hedonic goal, because these options are inferred to taste better and thus better fulfill the hedonic objective. Our hypothesis was that participants in the hedonic condition would show a stronger momentary activation of unhealthy = tasty associations. Behaviorally, this should translate into slower reaction times and less efficient response paths (higher MAD) for healthy relative to unhealthy food stimuli.

### Participants

We recruited 200 UK participants on Prolific, who were paid £1 for a task estimated to take 5 min. One participant did not deliver completed data and was removed from the dataset. Participants were on average 41.05 years old (*SD*_Age_ = 14.84; 69% female). All participants were screened on use of a desktop computer with an external mouse and on being right-handed. All participants provided informed consent prior to participation.

### Method

We manipulated goal orientation to influence the salience of UTI as in Raghunathan et al. (2006). Participants were divided randomly into two groups. In the utilitarian goal group (*n* = 100), participants were asked to think about craving healthy, nutritious food. Then they described what kind of food they were imagining, focusing on its nutritional value and how it contributed to their overall health goal. In the hedonic goal group (*n* = 99), participants were told to think about craving tasty, indulgent food. They described the food they were imagining, concentrating on its taste and how it satisfied their goal to indulge.

Next, participants participated in the UTI classification task, the explicit UTI scale, and demographics. As in Studies 1 and 2, the large majority of food items were classified as tasty in Study 3. In the utilitarian condition, 81% of healthy items and 88% of unhealthy items received a “tasty” response, while in the hedonic condition, 75% of healthy items and 88% of unhealthy items were classified as tasty.

To test our hypothesis that the hedonic goal manipulation activates temporary UTI beliefs, we preregistered the following analyses:

We examined RT difference scores between healthy and unhealthy food categorization, expecting that participants in the hedonic condition would be slower to categorize healthy foods relative to unhealthy foods, reflecting stronger UTI activation.

We analyzed MAD difference scores, hypothesizing that hedonic participants would show less efficient cursor trajectories (higher MAD) for healthy foods compared to the utilitarian group.

We also tested whether the goal manipulation affected taste classification responses (“tasty” vs. “not tasty”) using generalized linear mixed-effects models, predicting that hedonic participants would classify fewer healthy foods as tasty than utilitarian participants.

Lastly, we compared explicit UTI scale scores between groups in order to check whether the hedonic condition might show elevated explicit UTI in addition to the indirect measures. Because our focus is on the indirect measures, and the effect on the explicit measure may be less pronounced, we did not preregister this hypothesis.

### Preregistered analyses

#### Reaction times

**Fig. 3:**
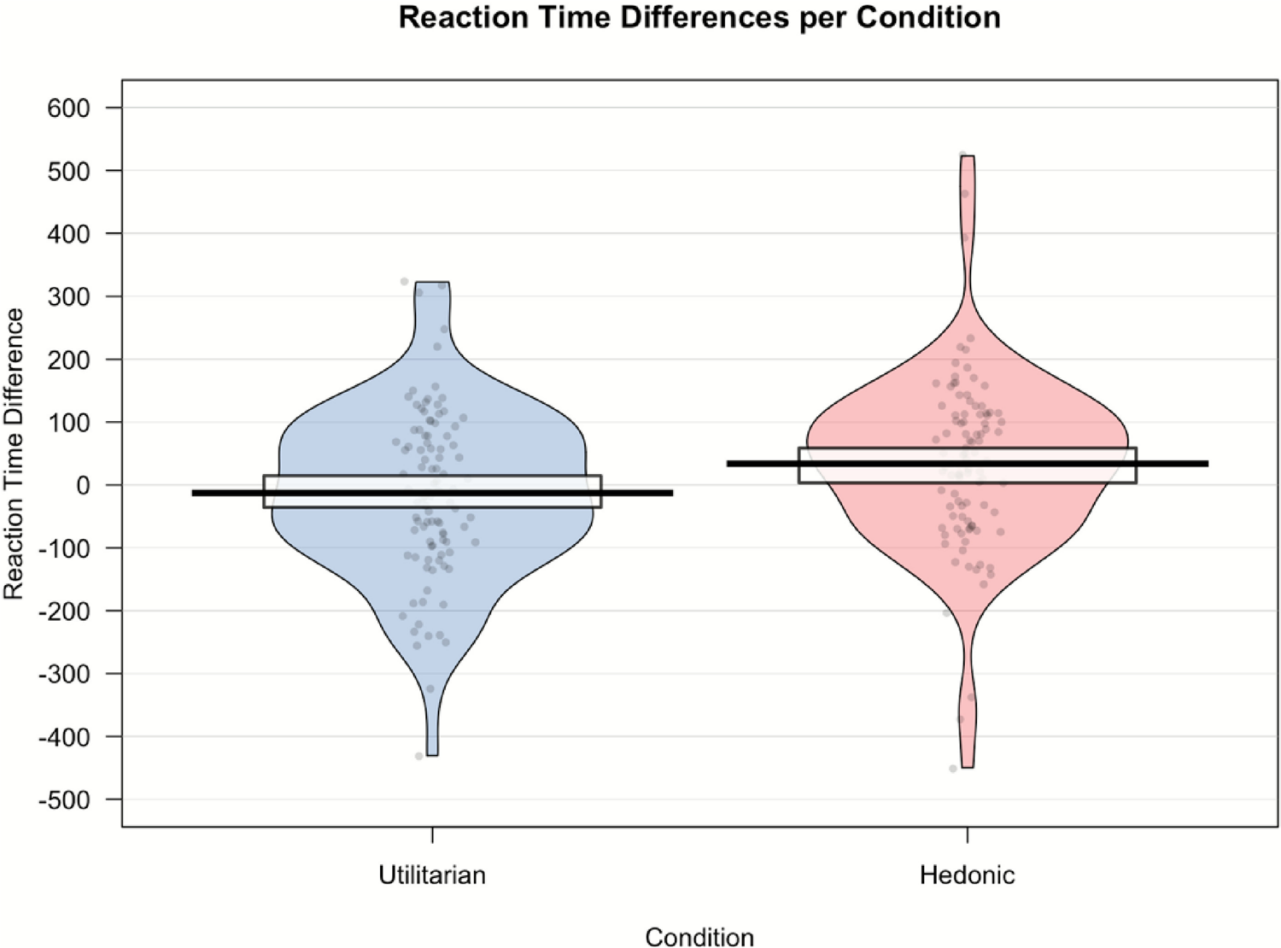
Pirate plot representing the reaction time differences for both conditions. The bold lines represent the group mean (with the rectangle representing the 95% confidence interval around the mean). The dots represent individual raw data points. The width of the colored areas indicates the density of the data

In investigating the difference between experimental conditions, an independent-samples *t*-test was conducted after removing outliers.^2^ Participants in the utilitarian condition had a negative mean reaction time difference score of *M*_RT_ = −12.90 (*SD*_RT_ = 135), indicating that they were faster categorizing healthy food items than unhealthy food items. Participants in the hedonic condition had a positive reaction time difference score mean of *M*_RT_ = 33.90 (*SD*_RT_ = 142), indicating that they were slower to categorize healthy food items than unhealthy food items. The *t*-test revealed a significant difference between the groups, *t*(196.7) = −2.37, *p* = .019, *d* = .34, 95% CI = [.06, .62]. See Fig. 3 for a visual representation of the data.

#### MAD

**Fig. 4:**
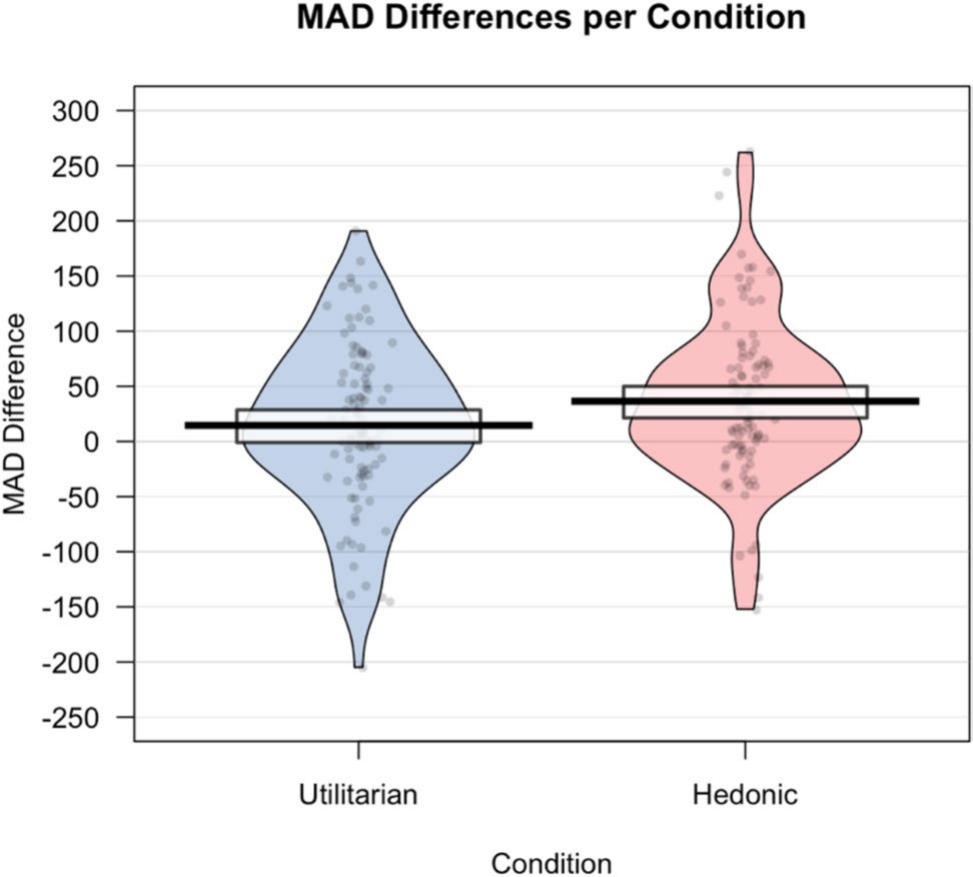
Pirate plot representing the MAD differences for both conditions. The bold lines represent the group mean (with the rectangle representing the 95% confidence interval around the mean). The dots represent individual raw data points. The width of the colored areas indicates the density of the data

**Fig. 5:**
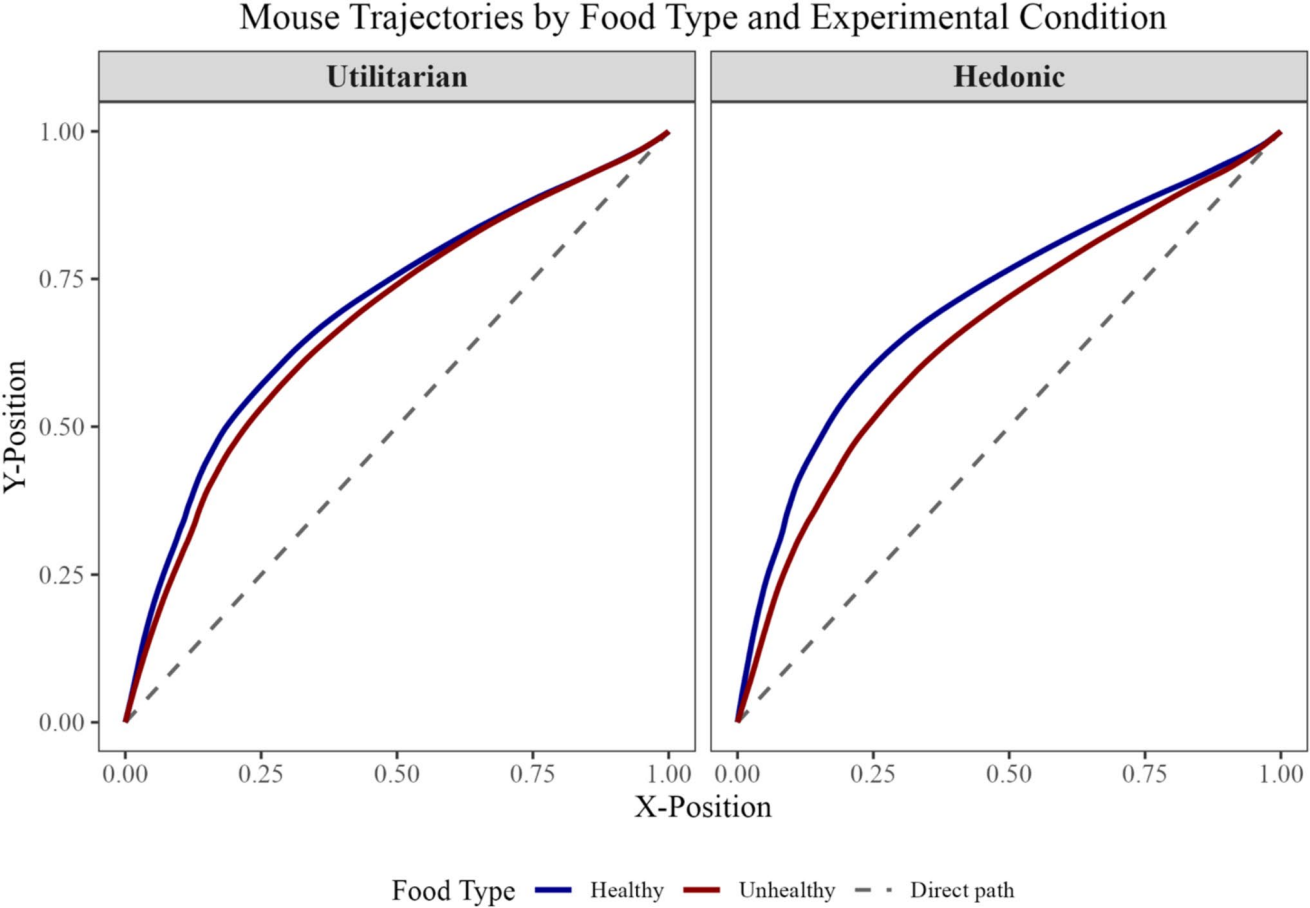
A visual representation of average trajectories for healthy and unhealthy food items in the utilitarian and the hedonic conditions

We found a similar effect in the MAD difference score, *t*(196.8) = −2.05, *p* = .042, *d* = .29, 95% CI = [.01, .57]. Participants in the hedonic condition were less efficient in categorizing healthy food images (*M*_MAD_ = 36.47, *SD*_MAD_ = 74.57) than participants in the utilitarian condition (*M*_MAD_ = 14.57, *SD*_MAD = _76.20). See Fig. 4 for a visual representation of the data and Fig. 5 for a visual representation of the trajectories in both conditions.

#### Classification responses

**Fig. 6:**
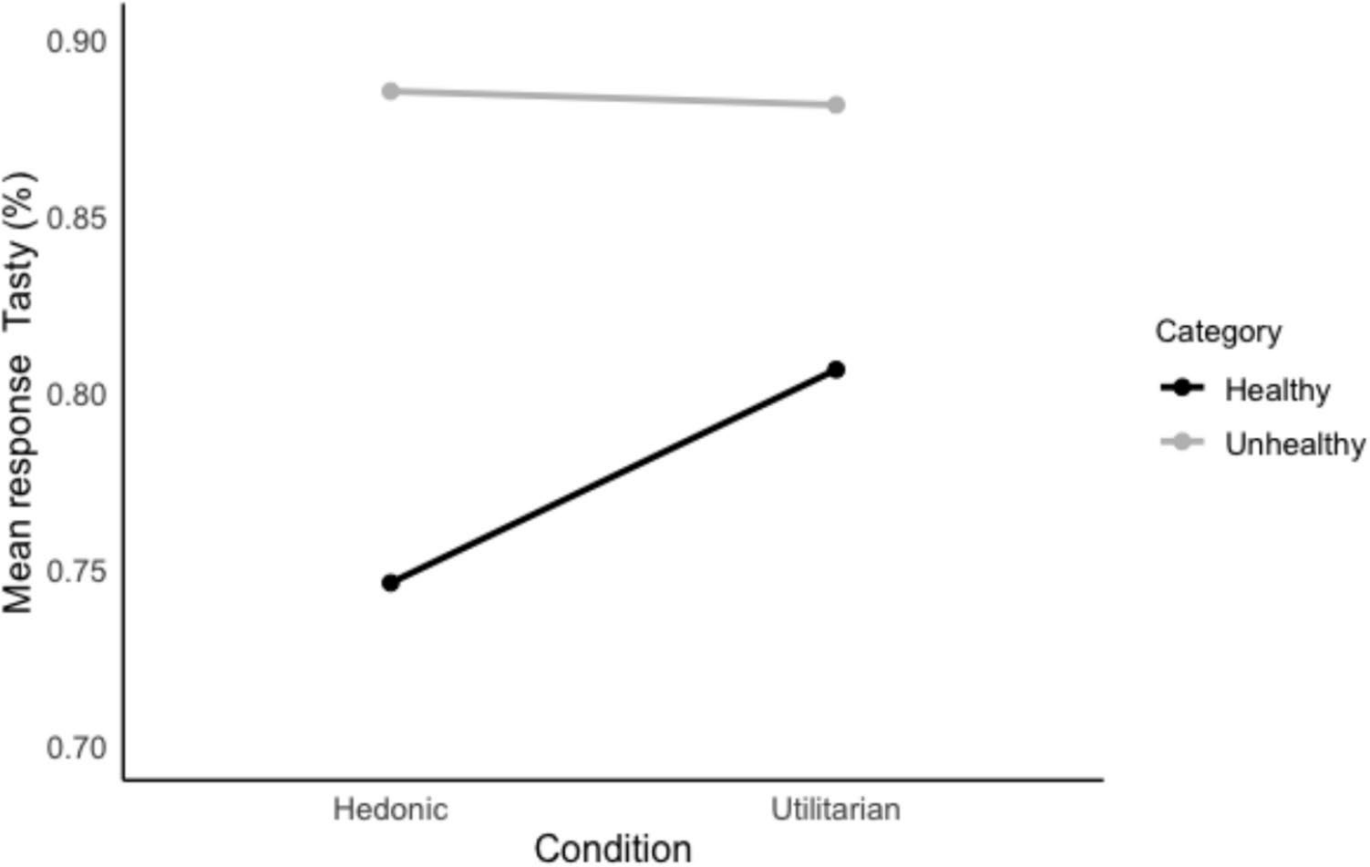
Overview of categorization responses per food category and condition

We examined whether the manipulation influenced the taste classifications. A generalized linear mixed model with classification response as dependent variable (0 = “not tasty,” 1 = “tasty”) and condition (0 = utilitarian, 1 = hedonic), food category (0 = healthy, 1 = unhealthy), and their interaction effect as independent variables was conducted. Age, gender, and education were added as covariates. Participant ID and food image (because images were shown multiple times) were added as random effects. We found a significant interaction effect between condition and food category, such that participants in the hedonic condition categorized fewer healthy food items as tasty compared to participants in the utilitarian condition, β = .49, *p* < .001 (Fig. 6).

#### Explicit UTI scale

Although the hedonic group showed a slightly higher mean explicit UTI score (*M* = 3.17, *SD* = 1.65) than the utilitarian group (*M* = 2.74, *SD* = 1.63), this difference was not statistically significant, *t*(197) = −1.84, *p* = .067, *d* = 0.26, 95% CI = [−0.02, 0.54].

### Discussion

Study 3 demonstrated that the mouse-tracking classification task is sensitive to an experimental manipulation of goal orientation, supporting its suitability for capturing temporary changes in UTI activation. Consistent with our hypotheses, participants primed with a hedonic goal exhibited slower reaction times and less efficient cursor trajectories (higher MAD) when categorizing healthy food items compared to those in the utilitarian condition. This pattern suggests that emphasizing indulgence increases cognitive conflict or hesitation when processing healthy foods, reflecting a stronger momentary activation of UTI beliefs.

Moreover, the hedonic manipulation influenced participants’ taste classifications: those in the hedonic condition were less likely to rate healthy foods as tasty, aligning with the idea that hedonic goals amplify the association between unhealthiness and tastiness. This behavioral shift further validates the task’s sensitivity to contextual cues shaping food evaluation.

Interestingly, the explicit UTI scale did not show a statistically significant difference between groups, indicating that these short-term contextual effects may be better captured by dynamic behavioral measures than by more stable self-report assessments. This divergence shows the complementary value of implicit tasks in studying transient cognitive biases that fluctuate with situational goals.

For exploratory purposes, we also fit trial-level mixed-effects models with condition, food type, and taste classification as fixed factors. These models did not reveal any additional theoretically interpretable interactions; full results are reported in Appendix C.

Overall, these findings extend previous work by demonstrating that the UTI classification task can detect experimentally induced fluctuations in food-related intuitions, making it a valuable research tool for investigating the mechanisms underlying food choice and decision-making in varied contexts.

## General discussion

In three studies, we validated a behavioral mouse-tracking task as a process-based index of the UTI. This lay belief, trading taste for health in food, has been implicated in influencing food choices and dietary habits. The results of Study 1 and Study 2 showed a significant correlation between participants’ explicit UTI scores and their performance on the classification task, as indicated by reaction times and, especially, mouse-tracking outcomes. The findings suggest that individuals with stronger UTI beliefs exhibit slower, less efficient taste categorization of healthy (vs. unhealthy) food items. Study 2 adds further to the content validity by showing that the classification task is also predictive of general diet quality. Study 3 introduced an experimental manipulation of goal orientation to examine the malleability of UTI beliefs. Participants primed with a hedonic goal exhibited stronger adherence to the UTI, as evidenced by slower reaction times and less efficient categorization of healthy (vs. unhealthy) food items. This shows the added value of this classification task over self-report scales, which were less sensitive to context effects.

This behavioral task, based on mouse-tracking, feels more natural than the IAT and can reveal more of the decision process. Moreover, it can be easily adapted to measure other constructs that are of interest to (consumer) researchers, to pick up the influence of contextual factors, such as differences in need states (e.g., being hungry or thirsty), goal activation (e.g., hedonic vs. functional goal), or the (consumption) environment, as well as the efficacy of behavioral interventions and communication strategies. For instance, it can be utilized to examine the impact of interventions on individuals’ associations with brands or organizations (Saint Clair & Cunha, 2024) or the perceived sustainability of products and packaging (Sokolova et al., 2023). To that end, we provide a blank template of the task that can be edited to fit the needs of researchers accordingly. The template can be found on https://osf.io/qej3k/overview.

In addition to this methodological contribution, we also add to the understanding of the nature of the UTI itself. Using the dimensions of taste and healthiness as categorization criteria for food results in four cells: healthy/tasty, healthy/not tasty, unhealthy/tasty, and unhealthy/not tasty. We are the first to disentangle the UTI in light hereof. In our studies, participants with lower and higher UTI beliefs did not behave differently when classifying unhealthy food items in terms of taste. However, a stark contrast could be seen in how they responded to healthy food items. As could be expected, participants with higher UTI scores demonstrated slower and less efficient categorization of healthy foods and categorized them as less tasty overall. This seems to be in line with the items of the commonly used explicit measurement of UTI (e.g., “Healthy food is less tasty”), which in hindsight appear to be tapping more into the perception of healthy foods than unhealthy food. Our data therefore suggest that, in practice, individual differences in UTI may manifest primarily as a reluctance to view healthy foods as tasty, rather than as unusually positive evaluations of unhealthy foods. Future work could examine this asymmetry more directly, for instance by including foods that vary independently in healthiness and tastiness, to test whether a “healthy = not tasty” component better captures the operative belief structure.

A major strength of this research is the consistent replication of findings across multiple studies, including a conceptual replication with a different set of stimuli and a varied experimental design. This robustness increases confidence in the validity and reliability of the mouse-tracking classification task as a measure of UTI. The use of multiple complementary measures, reaction time, maximum absolute deviation, and sample entropy, allowed for a nuanced understanding of decision dynamics, capturing not only overt behavior but also the underlying cognitive conflict.

Despite these strengths, all participants were adults recruited online, primarily from Western, educated populations, which limits the generalizability to more diverse cultural or age groups. This does offer a promising avenue for future research. This paradigm could be tested in younger populations, such as children and adolescents, where early interventions to modify food-related intuitions may have long-lasting impacts on diet and health. Studying UTI in children is especially important because early taste preferences and health beliefs shape lifelong eating habits and risk for diet-related diseases (e.g., Skinner et al., 2002). Previous work has focused on finding child-appropriate measurement tools for trait UTI (D’hondt & Briers, 2024), but an adaptation of this task to be child-friendly could open new avenues for developmental research. A second limitation is our reliance on a self-reported food frequency questionnaire (FFQ) as the “downstream” behavioral outcome. FFQs remain the field’s benchmark for assessing habitual diet and have repeatedly served as a meaningful consequence of UTI beliefs (e.g., Briers et al., 2020; Cooremans et al., 2017); for establishing that our classification task relates to stable eating patterns, they are therefore appropriate. At the same time, FFQs are retrospective, vulnerable to recall bias, and insensitive to short-term, experimentally induced shifts. One might suggest adding an immediate behavioral choice task, yet such tasks are themselves prone to carryover effects from preceding food-related manipulations (Barakchian et al., 2021). Because the classification task could inadvertently prime particular choices, a laboratory buffet or snack selection administered immediately afterward might conflate task-induced bias with genuine preference. For the present aims, linking UTI to enduring dietary habits, the FFQ offered a cleaner measure. Future studies could complement this approach by including objective or real-time outcomes (e.g., digital purchase logs, ecological momentary assessment, or food choices collected in a separate session) to determine whether the mouse-tracking indices also predict immediate behavior, without the confound of task-ordering effects. A further limitation concerns the analytical granularity of our mouse-tracking data. We relied on summary indices such as MAD, a common and defensible choice in the literature (Cummins & De Houwer, 2021), because these averages were sufficient to test our preregistered hypotheses about overall conflict and efficiency. Nevertheless, this strategy collapses the rich moment-to-moment information embedded in each trajectory. Mouse-tracking can, for example, be decomposed into velocity or acceleration profiles in 50–100 ms bins, analyzed with functional principal components analysis, or modeled as a full time series. Such fine-grained approaches are better suited to questions about when conflict first emerges or how it evolves over the course of a movement. While that level of complexity was unnecessary for the present aims, future research could exploit these advanced techniques to address more complex hypotheses and to capitalize on the full temporal richness of mouse-tracking data.

In conclusion, this research contributes to the literature on food choice by introducing a novel behavioral mouse-tracking task as a process-based index of the UTI. Our findings suggest that this task offers added value above existing self-report scales, demonstrating sensitivity to situational influences and capturing dynamic decision processes. This makes it a particularly suitable tool for both basic research and applied interventions aimed at better understanding and ultimately changing unhealthy eating behaviors.

## Data Availability

The preregistrations, stimuli, and data analysis code for all studies are available on the Open Science Framework (https://osf.io/qej3k/overview).

## Footnotes

1 See Scherbaum and Kieslich (2018) for a discussion on dynamic versus static starting procedures in which they recommend the dynamic procedure; however, we also replicate these results with a static starting procedure in Appendix A.

2 We preregistered to remove reaction time outliers larger than three standard deviations of the mean, which was 1,247 ms before exclusions. However, this only removed one extreme outlier (707,684 ms) and left another outlier (23,516 ms) that skewed the results. We removed this outlier by adjusting the threshold to two standard deviations. Only these two values were removed as a result.

## Code availability

Software files are available on https://osf.io/qej3k/overview.
